# Plant size influences abundance of floral visitors and biomass allocation for the cushion plant *Thylacospermum caespitosum* under an extreme alpine environment

**DOI:** 10.1002/ece3.5147

**Published:** 2019-04-10

**Authors:** Ruiming Zhao, Hua Zhang, Lizhe An

**Affiliations:** ^1^ MOE Key Laboratory of Cell Activities and Stress Adaptations School of Life Sciences Lanzhou University Lanzhou China; ^2^ The College of Forestry Beijing Forestry University Beijing China

**Keywords:** cushion plants, flies, ontogeny, pollination, reproductive allocation, seed set

## Abstract

Variation in size may influence the abundance of visitors and reproductive allocation for cushion plants in the extreme alpine environments. To assess effects of plant size on the abundance of main visitors and reproductive allocation in *Thylacospermum caespitosum* populations at two altitudes, the abundance of the visitors, visiting frequency, total number of flowers, number of fruits, number of unseeded flowers, and reproductive allocation were investigated during the period of reproductive growth. Concurrently, the effects of plant size on the visitors' contributions to fruit setting rate were assessed by a bagging experiment. Our results showed that flies (*Musca domestica* and *Dasyphora asiatica*) were the main pollinating insects of *T. caespitosum*, and they could obvious facilitate (*p* < 0.05) the fruit setting rate of this cushion plant. Seed set and floral visitation were significantly influenced (*p* < 0.001) by plant size. Moreover, the reproductive allocation and fruit setting rate of *T. caespitosum* was influenced (*p* < 0.001) by plant size. More biomass was allocated to reproduction in plants of greater diameter. There is an increase in reproductive success (increases of fruit number with increase in plant size) in relation to plant size. In conclusion, the extent of *M. domestica* and *D. asiatica* to facilitate the fruit setting rate mainly depended on the size of *T. caespitosum*. Size‐dependent reproductive allocation occurred in *T. caespitosum* and was the chief factor affecting the contribution of flies to fruit setting rate. These traits reflect reproductive fitness of *T. caespitosum* related to plant size in extreme alpine environments.

## 1 INTRODUCTION

Insect visitors can enhance pollination and fruit set of plants, and plants can offer rewards for visitor insects (Bailes, Ollerton, Pattrick, & Glover, 2015). Most flowering plants need visitors to complete their reproductive cycle (Zhu, Yang, & Li, 2013), and attracting multiple visitors may benefit plants by increasing seed set. Visitors usually get rewards from flowering plants, of which food resources (such as nectar, pollen, oil, or other substances) are the most common rewards for the visitor (Simpson & Neff, 1983). Additionally, some flowers act as a shelter for floral visitors, offering protection from predators and cold climate (Sapir, Shmida, & Ne'eman, G., 2006; Seymour, White, & Gibernau, 2003; Zhu et al., 2013). In fact, there are flexible relationships between the majority of flowering plants and their visitors (Waser, Chittka, Price, Williams, & Ollerton, 1996). These flexible relationships might be related to the characteristics and habits of different taxonomic/functional groups and the local ecological context (Fenster, Armbruster, Wilson, Dudash, & Thomson, 2004; Ollerton, Killick, Lamborn, Watts, & Whiston, 2007). This relationship between plants and insect visitors is well known in agricultural crops (Deguines, Julliard, Flores, & Fontaine, 2016; Garibaldi et al., 2015; Klein, Steffan‐Dewenter, Buchori, & Tscharntke, 2002; Orford, Vaughan, & Memmott, 2015; Parsche, Fründ, & Tscharntke, 2011) and some wild plants (such as catchfly, invasive species, and other species; Brown, Lynch, & Zilberman, 2002; Flanagan, Mitchell, & Karron, 2011; Lance, Bailey, Lindsay, & Cobb, 2017; Mustajärvi, Siikamäki, Rytkönen, & Lammi, 2001; Yang, Ferrari, & Shea, 2010), which from agricultural and wild ecosystem. However, relationships between cushion plants and insect visitors in extreme alpine environments are not well understood.

Traditionally, bees, hoverflies, and butterflies are frequently studied as pollinators in agricultural and conservation research; wild and managed bees are well documented as effective pollinators in agricultural and other ecosystems (Orford et al., 2015). However, many flies, bumblebees, and Lepidoptera species are known contributors to pollination in alpine ecosystems (Arnold, Savolainen, & Chittka, 2009; Galen, 1996). In the subnival belt, the high altitude, low temperatures, overcast conditions, short growing season, unstable substrate, intense radiation, and relatively unpredictable weather and high winds are all challenging for insect visitors (Cavieres, Quiroz, Molina‐Montenegro, Muñoz, & Pauchard, 2005; Körner, 2003). Thus, the levels of diversity and activity of insect visitors are reduced due to the harsh climatic conditions in alpine ecosystems, and pollination rates are accordingly inherently low (Torres‐Díaz et al., 2011). These climatic characteristics also progressively reduce pollen availability in alpine plants (Bingham & Orthner, 1998). Alpine plants have developed solutions to deal with low visitor rates and reduction in pollen availability, such as self‐compatible (Liu et al., 2017; Sosenski, Ramos, Domínguez, Boege, & Fornoni, 2017) plants are an evolutionary solution for alpine and arctic plants to deal with low visitor numbers; thus, they will face lower risk of extinction due to low supply of pollen from compatible plants (García‐Camacho & Totland, 2009; Muñoz & Arroyo, 2006). Alpine plants in the subnival belt demonstrate a high frequency of asexual reproduction (Morgan, Wilson, & Knight, 2005; Reid, Hooper, Molenda, & Lortie, 2014). This reproductive strategy benefits alpine plants in harsh climatic conditions with low diversity and activities of pollinators (Milla, Giménez‐Benavides, Escudero, & Reich, 2009). Theoretical and empirical studies have predicted that severe environmental conditions (climatic conditions and insufficient visitors) lead to greater levels of asexual reproduction compared to lower alpine elevations, where high metabolic costs are invested in sexual reproduction relative to asexual reproduction (Chen, Li, Yang, & Sun, 2017; Schuster & Longton, 1983, Stark, Mishler, & McLetchie, 2000). Alpine plants can also compensate for the scarcity of visitors in alpine habitats by increasing the flowering phase and flower longevity (Blionis & Vokou, 2002; Fabbro & Körner, 2004).

Although alpine plants benefit from self‐compatible, asexual reproduction, longer flowering phase, and flower longevity to compensate for the low diversity and activity of pollinators, many alpine species are strongly dependent on scarce insect visitors to increase their seed set. Alpine flowers (*Saxifraga oppositifolia*,* Dryas integrifolia*, and *Salix arctica*) that are frequently visited by insects (pollinated primarily by flies and bumblebees) are totally or partially dependent on them for seed set (Kevan, 1972; Peeters & Totland, 1999). Here, we investigated whether and how strongly floral visitors influenced seed set in an alpine plant species, and what plant traits determined visitor abundance. High allocation to the production of reproductive structures also dictates the extent to which floral visitors pollinate flowers (Campbell & Halama, 1993). Producing (more nectar) may increase visitation rates as well as pollination success, and these in turn result in greater fruit and seed production (Mattila & Kuitunen, 2000; Sletvold, Tye, & Ågren, 2017). Moreover, environmental conditions can affect reproductive allocation patterns in plants. Variation in environmental conditions during reproduction may result in differences in ovule number, germination rates, growth rates of pollen tubes, and seed production (Jennersten, 1991; Young & Stanton, 1990). Alpine plants, hereafter cushion plants, tend toward a highly compact growth form that slowly (ca. 0–7 cm per year) grows along the ground, forming dense mats of vegetation. These slow‐growing species often have very long‐lived leaves (many cushions can grow for centuries or persist for additional centuries; Chen et al., 2017; Forbis & Doak, 2004; le Roux & McGeoch, 2004; Molau, 1997). Previous studies have shown that some cushion species may need 20 years of vegetative growth in order to begin reproduction for the remainder of their life history (Molau, 1997; Morris & Doak, 1998). These characteristics indirectly reflect that plant size may influence reproductive allocation in cushion plants. Thus, studies examining reproductive allocation need to consider the potential effect of size‐related variation in cushion species. After all, study of size dependence of fitness components is usually the only way to apply life history theory predictions to most herbaceous perennials due to the difficulty in evaluating the age of these plants except where long‐term demographic studies are conducted (Shabir, Nawchoo, & Wani, 2017).

Size‐dependent variation in reproductive allocation is a common phenomenon in many plant species (Samson & Werk, 1986). Plant size is widely used in the prediction of current and future reproductive output, and the relative advantages or disadvantages of reproducing at different sizes (Stearns, 1992; Sletvold, 2002). Ollerton and Lack (1998) indicated that plant size not only directly influences individual plant fecundity but also can indirectly affect reproductive output. The largest plants are commonly the most fecund, and size is closely correlated with total flower production in populations (Weiner, 1988; Herrera, 1993). In fact, the effect of plant size on reproductive output has been broadly discussed in extreme environments (Aarssen, 2015), such as desert and saline environments (Waller, 1988; Aguilar, Ashworth, Galetto, & Aizen, 2006; del Carmen Mandujano, Carrillo‐Angeles, Martínez‐Peralta, & Golubov, 2010; Benassi et al., 2011; Chacoff et al., 2012; Paasch, Mishler, Nosratinia, Stark, & Fisher, 2015; Salguero‐Gómez et al., 2016). Moreover, although alpine plants benefit from asexual reproduction to compensate for the low diversity and activity of pollinators, many cushion species are entirely dependent on sexual reproduction (Chen et al., 2017). *Thylacospermum caespitosum* (Caryophyllaceae) represents this type of cushion species. But plant size influences the abundance of floral visitors and biomass allocation for the cushion plant under an extreme alpine environment remains unknown. Here, we investigated whether the fruit setting rate promoted by visitors was related to the size of *T. caespitosum*. We also evaluated the effects of plant size on the abundance of the main visitors, visiting frequency, total number of flowers, number of fruits, number of unseeded flowers, reproductive biomass ratio, stem‐leaf biomass ratio, root biomass ratio, and fruit setting rate. We aimed to answer the following questions: (a) Who are the main visitors of *T. caespitosum*? (b) How do cushion plants (*T. caespitosum*) in extreme environments reproduce as they increase in size? And (c) how do flower visitors interact with *T. caespitosum* to influence fitness?

## 2 METHODS

### 2.1 Study site and study species

**Figure 1:**
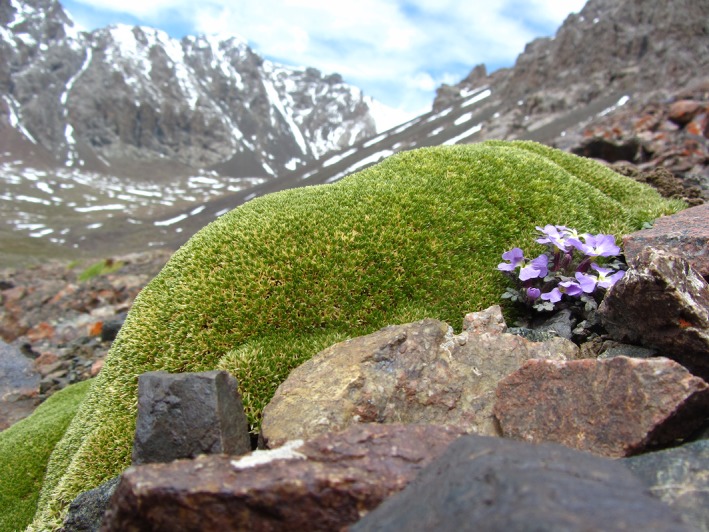
Flank view of a *Thylacospermum caespitosum* in the eastern Tianshan Mountains, China

The study site was situated in the headwater region of the Urumqi River in the eastern Tianshan Mountains, China. The site is part of the Xinjiang Uygur Autonomous Region. The elevation varies from about 4,100 m to 4,300 m. At the study site, mean annual temperature ranges from ca. 5 during the day and −4°C at night; however, temperature as low as −10 may occur during the vegetative growth season (Liu et al., 2017). As Figure 1 shown, *T. caespitosum* is a perennial plant that has a woody taproot and forms very dense and solid cushions (Dvorský et al., 2013). *Thylacospermum caespitosum* is one of the most representative high‐alpine cushion plants in Asian high mountain regions. It is distributed along rocky slopes and crevices from 3,600–6,000 m asl. *Thylacospermum caespitosum* is found in China (Gansu, Qinghai, Sichuan, Xinjiang, and Xizang), India, Kazakhstan, Kyrgyzstan, Nepal, and Sikkim (Flora of China Editorial Committee, 1999). *Thylacospermum caespitosum* plants have been studied to understand their ameliorating effects on the harsh environments they inhabit (extreme altitude and dry conditions) and their role as nurse plants for other plant species in alpine ecosystems (Bello et al., 2011; Chen et al., 2015; Dvorský et al., 2013; Michalet et al., 2016).

In the study region, *T. caespitosum* was distributed in the range of 3,500–4,100 m elevation. Meantime *T. caespitosum* is foundation species of cushion vegetation in this region. *Thylacospermum caespitosum* exhibited four main flowering stages as follows (data from our observations in 2015): bud differentiation stage (10 May–20 May), initial flowering stage (20 May–10 June), full‐bloom stage (10 June–10 July), and flower fading stage (after 10 July). Experiments were conducted from May 2016 to October 2016 at the study site.

### 2.2 Effects of plant size on insect visitation rate

Two populations of *T. caespitosum* were used: a low elevation population (3,600 m) and a high elevation population (3,900 m). The two populations were about 5 km apart. In order to assess the effects of plant size on insect visitation, 20 flowering plants in each of the two populations were selected to monitor floral visitors. In each population, the selected plants were assigned to the following four plant size classes (each size have five flowering plants): R1: diameter of the plant ranged from 10 to 20 cm; R2: diameter of the plant ranged from 20 to 40 cm; R3: diameter of the plant ranged from 40 to 60 cm; and R4: diameter of the plant ranged from 60 to 80 cm. Floral visitor observation was performed for 5 days on every selected plant during full‐bloom stage (according to our observations, the full‐bloom stage of *T. caespitosum* lasted for 4–6 days). Visitor observations were carried out between 10:00 and 14:00, which was the peak time for visitors in the extreme alpine environment. Each observation on each flowering plant lasted for 30 min one day. The species of visiting insects, number of visiting insects, and visiting frequency were recorded for both populations. The different days for one plant were averaged for one replicate. Thus there are 5 replicates at per size class, and 20 plants per population (*N* = 40).

### 2.3 Effects of plant size on reproductive allocation

The selected plants used to determine visitation rate were used to count flowers (during flowering) and fruits (after flowering) of *T. caespitosum* by using circular PVC (polyvinyl chloride) piles with 10 cm radius (157 cm^2^). The centers of PVC piles coincided with the cushion plant. With these data, we could measure the total number of flowers, number of fruits, and number of unseeded flowers. At the same time, all of the above plants were collected at the end of the growing season to estimate biomass allocation (g). All samples were dried at 70°C for 48 hr and weighed (Ploschuk, Slafer, & Ravetta, 2005). Plant biomass was divided into reproductive biomass (all reproductive structures), stem‐leaf weight biomass, and root biomass.

### 2.4 Effects of plant size on visitors' contributions to fruit setting rate

Sixty flowering plants in each of the two populations were selected to assess the effect of visitors on fruit setting rate of *T. caespitosum*. In each population, the selected plants were assigned four classes as shown in Section 2.2. For each population, we randomly selected 15 plants in each of the four size classes (*N* = 60 for each population) and assigned them to one of two treatments: open pollination or exclusion of insect visitors. Twenty flowers (10 flowers for open pollination and 10 for exclusion of insect visitors on each plant) were randomly selected per plant, and we randomly divided the flowers into three sampling sessions. Thus, the 15 plants in each size class were divided into three groups (each group included five plants, with a total of 50 flowers marked as open pollination and 50 as exclusion of insect visitors). One group represented one repetition. Flowers in the open pollination treatment had no interference with regard to floral visitors, whereas flowers in the exclusion treatment were covered with nylon mesh bags to prevent visitation. We collected all the fruits before dehiscence to count fruit setting rate at harvest time. To evaluate the net outcome of the association between visitors and fruit setting rate of plants, we used the relative interaction index RII (Armas, Ordiales, & Pugnaire, 2004). RII*_visitor_* was used to quantify the intensity of the direct visitors' effects on fruit setting rate of *T. caespitosum*. RII*_visitor_* is bounded between −1 and 1, with negative values indicating a negative influence of visitors on fruit setting rate, positive values indicating positive influence, and larger absolute values indicating stronger influence of the visitors.

$${\text{RII}}_{\mathrm{visitor}}=\frac{V1-V2}{V1+V2}$$

The variables are as follows: V1, mean fruit setting rate with open pollination; V2, mean fruit setting rate with exclusion of floral visitors.

### 2.5 Statistical analysis

All experimental results were presented as the mean ± standard error. To test the effect of plant size on abundance of main visitors and reproductive allocation, all parameters (number of flies, visiting frequency, total number of flowers, number of fruits, number of unseeded flowers, flower weight ratio, stem‐leaf weight ratio, root weight ratio, fruit setting rate, and RII*_visitor_*) were, respectively, evaluated by the generalized linear mixed model (GLMM), where plant number and population were random factors and plant size was fixed factor. Poisson error structure was used in the model. Analyses were performed using the MASS package of R 3.5.1 (R Development Core Team, 2011). Moreover, a paired sample test was used to evaluate differences in fruit setting rate between V1 and V2, and statistical analyses were performed using SPSS ver. 16.0 (SPSS, Chicago, Illinois, USA).

## 3 RESULTS

**Figure 2:**
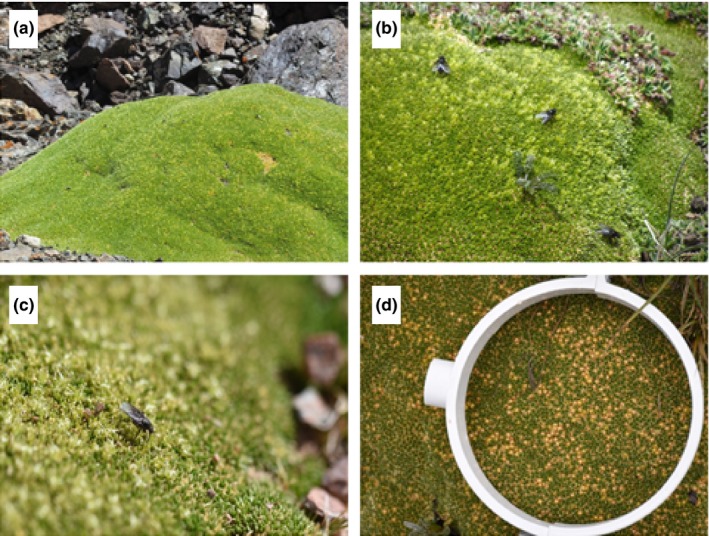
*Thylacospermum caespitosum* (a) and its visitors (b and c) and fruit (d)

**Figure 3:**
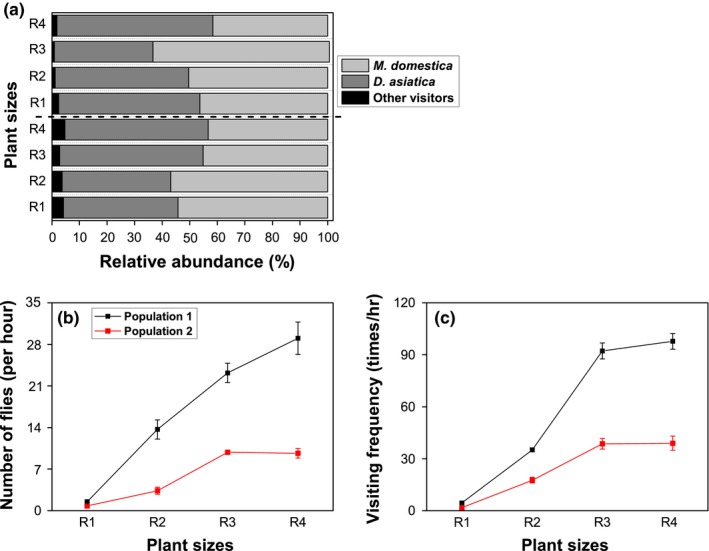
Changes in relative abundance of visitors (a), fly abundance (b), and visiting frequency (c) in relation to different sizes of *Thylacospermum caespitosum* plants at two different altitudes. Error bars indicate the standard error of the means (*n* = 5)

**Figure 4:**
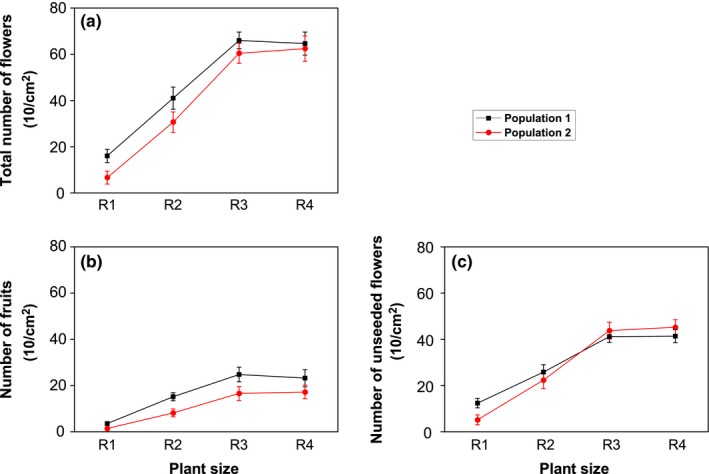
Change in number of flowers (100 cm^2^), number of fruits (100 cm^2^), and number of unseeded flowers (100 cm^2^) in relation to different sizes of *Thylacospermum caespitosum* plants at two different altitudes. Error bars indicate the standard error of the means (*n* = 5)

Results of field investigations showed that flies (*Musca domestica* and *Dasyphora asiatica*) were the main flower‐visiting insects for *T. caespitosum* (Figures 2 and 3a). Relative abundance of flies was >93% of visitors for all plant sizes of the two populations (Figure 3a). Although *T. caespitosum* has small flowers (about 2.0–3.5 mm; Figure 2c), the flowers were abundant once in reproductive stage. For example, the number of flowers reached 66.0 and 64.6 per 100 cm^2^ for the two largest plant size classes in the lower altitude population and 60.4 and 62.4 per 100 cm^2^ in the same size classes in the higher altitude population (Figures 2d and 4).

As shown in Table 1, number of flies and visiting frequency of *T. caespitosum* were dramatically (*p* < 0.001) influenced by plant size. For the lower altitude population, fly abundance and visited flowers were increased significantly with larger plant size (Figure 3b,c). When compared with R1, number of flies and visiting frequency were increased by 8.0 (R2) times, 14.3 (R3) times, and 18.1 (R4) times in the low altitude population; and 6.8 (R2) times, 19.6 (R3) times, and 20.8 (R4) times in the lower altitude population, respectively (Figure 3b,c). Similar results were observed in the high altitude population. These results indicated that the size of *T. caespitosum* was one of the most important factors influencing the number of flies and visited flowers. The greater the plant size, the more flowers were visited by flies.

**Table 1:** Effects of plant size on abundance of main visitors and reproductive allocation in *Thylacospermum caespitosum*

Fixed effect variables	Model parameter			
	*β* ± *SE*	95% CI	*t*	*p*
Number of flies (hr)	0.90 ± 0.10	−0.00007 to 0.00003	8.89	<0.001
Visiting frequency (times hr)	2.53 ± 0.30	−0.830 to 0.031	8.36	<0.001
Total number of flowers (100 cm^2^)	0.45 ± 0.05	−1.972 to 1.658	8.32	<0.001
Number of fruits (100 cm^2^)	0.50 ± 0.09	−1.667 to 1.178	5.50	<0.001
Number of unseeded flowers (100 cm^2^)	0.43 ± 0.05	−2.088 to 2.360	8.43	<0.001
Flower weight ratio (%)	0.42 ± 0.08	−1.468 to 1.524	5.19	<0.001
Stem‐leaf weight ratio (%)	−0.01 ± 0.01	−1.733 to 1.877	−0.70	0.4867
Root weight ratio (%)	−0.02 ± 0.01	−3.069 to 2.200	−1.54	0.1307
V1 (%)	0.27 ± 0.06	−1.034 to 0.721	4.70	<0.001
V2 (%)	0.02 ± 0.05	−1.837 to 1.671	0.43	0.6724
RII*_visitor_*	0.54 ± 0.07	−1.119 to 0.672	8.01	<0.001

Plant size had a great effect on total number of flowers, number of fruits, and number of unseeded flowers of *T. caespitosum* (*p* < 0.001, Table 1). For the lower altitude population, total number of flowers, number of fruits, and number of unseeded flowers number increased significantly (*p* < 0.05) when compared with R1 (Figure 4). When compared with R1, the total number of flowers, number of fruits, and number of unseeded flowers increased by 1.6 (R2) times, 3.1 (R3) times, 3.0 (R4) times (Figure 4). Similar changes were observed in the high altitude population (Figure 4). These results indicated that the size of *T. caespitosum* influences total number of flowers, number of fruits, and number of unseeded flowers.

**Figure 5:**
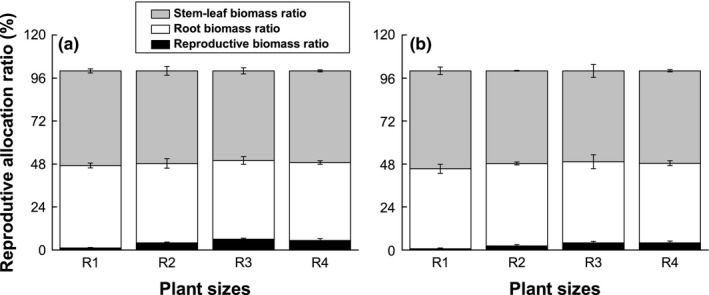
Changes in flower weight ratio, stem‐leaf weight ratio, and root weight ratio in relation to different sizes of *Thylacospermum caespitosum* plants at two different altitudes (a, low elevation and b, high elevation). Error bars indicate the standard error of the means (*n* = 5)

The reproductive biomass ratio of *T. caespitosum* was significantly affected (*p* < 0.001) by plant size, but stem‐leaf weight ratio and root weight ratio not be affected (*p* > 0.05, Table 1). For the lower altitude population of *T. caespitosum*, the flower biomass ratio was significantly increased (*p* < 0.05) by 2.3 (R2) times, 4.0 (R3) times, and 3.5 (R4) times compared to R1 (Figure 5). Similarly, flower weight ratio significantly increased (*p* < 0.05) by 2.4 (R2) times, 4.9 (R3) times, and 4.9 (R4) when compared with R1 for high altitude populations. These results indicated that reproductive allocation of *T. caespitosum* was clearly influenced by plant size.

**Figure 6:**
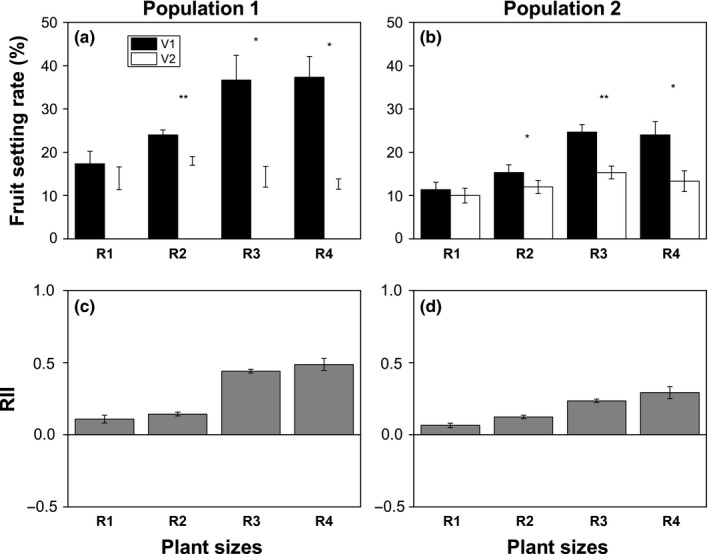
Fruit setting rate during open pollination (V1) and isolation from insect visitors (V2) in two populations (a, low elevation and b, high elevation): mean relative interaction index (RII) of visitor effects on two pollinations of *Thylacospermum caespitosum* (c, low elevation and d, high elevation). Error bars indicate the standard error of the means (*n* = 3). ^*^represents differences among two populations, ^*^ *p* < 0.05, ^**^ *p* < 0.01

Fruit setting rate during open pollination (V1) was significantly affected (*p* < 0.001) by size of *T. caespitosum* plants (*p* < 0.001, Table 1). For the lower altitude population of *T. caespitosum*, V1 was significantly increased (*p* < 0.05) by 2.1 (R3) times and 2.2 (R4) times compared with R1 (Figure 6a). Similarly, V1 was significantly increased (*p* < 0.05) by 2.2 (R3) times and 2.1 (R4) times when compared with R1 for the high altitude population. However, fruit setting rate during the exclusion of insect visitors (V2) was not affected by plant size in the two populations (*p* > 0.05, Table 1). V1 was significantly higher (*p* < 0.05) than V2 except for R1 in the low and high elevation populations (Figure 6a,b). Moreover, plant size has a great effect on RII of *T. caespitosum* (*p* < 0.001, Table 1). RII increased with the increase in plant size, with R3 and R4 significantly higher (*p* < 0.05) than R1 and R2 (Figure 6c,d). These results indicated that flies may facilitate fruit setting in *T. caespitosum*, which was especially obvious in larger plants.

## 4 DISCUSSION

Insect visitor diversity, abundance, and activity decrease dramatically in extreme alpine environments relative to tropical (or lowland) regions due to harsh climatic conditions (Körner, 2003; Medan et al., 2002). Scarcity of insect visitors adversely affects plant reproduction (Kuijper & Pen, 2014). However, alpine flowers are frequently visited by insects and are totally or partially dependent on them for seed set (Kevan, 1972; Peeters & Totland, 1999). Our results indicated that flies were the major visiting insects for *T. caespitosum*, and they may promote fruit setting of this cushion plant (especially in larger plants; Figures 2, 3 and 6). But bees, hoverflies, and butterflies (taxa frequently studied as visitors in agricultural and conservation contexts) were not observed in our study. In fact, more researchers are realizing that the contributions of visitors other than bees (such as nonsyrphid Diptera) have been underexplored despite their potential to contribute to plant reproduction and stability in the face of environmental change (Rader et al., 2016; Tiusanen, Hebert, Schmidt, & Roslin, 2016). Lefebvre, Fontaine, Villemant, and Daugeron (2014) indicated that flies (flies represent more than 60% of all visitors, with 54% of them being Empidinae) widely replaced bees as the main flower visitors in a subalpine meadow in the French Alps, and among them the Empidinae might play a key role in pollination. Kevan (1972) and Peeters and Totland (1999) showed that alpine flowers most frequently visited by flies and bumblebees are totally or partially dependent on them for seed set.

We then ask the question, why were floral visitors predominantly flies? On one hand, nonsyrphid dipteran visitors are dominant at high altitudes and latitudes, including alpine and subarctic ecosystems where bees are less abundant, mainly due to the loss of habitat from anthropogenic land use change and intensification (Elberling & Olesen, 1999; Shrestha et al., 2016; Vanbergen et al., 2014; Williams & Osborne, 2009). The proportion of Muscidae species usually increases with altitude, but the proportions of Syrphidae and bumblebees decreases (Pont, 1993). On the other hand, the scarcity of flower visitors with long proboscises in the alpine ecosystem may have opened a niche for flies and put a selection premium (extra charges in selection process) on a longer proboscis and more shallow flowers (Elberling & Olesen, 1999). This may indirectly help flies become predominant floral visitors of *T. caespitosum*. Moreover, our results were consistent with the previous view that flies (Muscidae) are probably the most important flower visitors above the timberline and may facilitate (The greater the plant size, the more flowers were visited by flies and the higher the fruit setting rate in *T. caespitosum*) the pollination of alpine plants (Larson, Kevan, & Inouye, 2001; Orford et al., 2015). In fact, flies (and other nonbee taxa) often have broader temporal activity ranges and can provide pollination services at different times of the day compared with bees and in weather conditions when bees are unable to forage (Howlett, 2012; McCall & Primack, 1992; Rader, Edwards, Westcott, Cunningham, & Howlett, 2013). Rader et al. (2016) indicated that nonbee insect (including flies) visitors play a significant role in global crop production and potentially make pollination services more robust to changes in land use. Thus, we conclude that floral visitors with the capacity to pollinate *T. caespitosum* over a broader temporal range (primarily nonbee taxa) have a stronger influence on their reproductive fitness than those with a more restricted temporal range. This is significant because it demonstrates how abiotic conditions select for particular pollinators, and how this may have a cascading effect on a long‐lived plant's fitness. Seed set and floral visitation of *T. caespitosum* were influenced by plant size. One possible reason is that small plants have less investment in reproduction relative to large individuals. Plant size influences several important fitness components (flowering probability, reproductive allocation, and fecundity) of herbaceous perennial plants (Msndez & Karlsson, 2004). This size‐dependent reproductive allocation (large plants generally have large reproductive investment) is common in alpine plants (Sun et al., 2014). Our results support the presence of more flowers and greater reproductive allocation in larger *T. caespitosum* plants (Figures 3 and 4; Table 1). Large reproductive investment means more materials will be used to attract insects for pollination. Moreover, difficulty in allocating nonstructural carbohydrates (as a main reward to visitors) to reproduction exists in extreme conditions. Under harsh environments, nonstructural carbohydrates can help plants to survive (Chapin, Schulze, & Mooney, 1990; Dietze et al., 2014). Theses carbohydrates correlate with resistance to extreme conditions (Hartmann, Ziegler, Kolle, & Trumbore, 2013; O'Brien, Leuzinger, Philipson, Tay, & Hector, 2014; Sala, Woodruff, & Meinzer, 2012; Slewinski, 2012; Tattini, Gucci, Romani, Baldi, & Everard, 1996), where nonstructural carbohydrates accumulate in plant tissue for use in cryoprotection, desiccation protection, and maintenance of turgor pressure and ionic composition (Ögren, Nilsson, & Sundblad, 1997; Myers & Kitajima, 2007; Bansal & Germino, 2008, 2009; Bansal, Reinhardt, & Germino, 2011). It is not cost‐effective for visitors to visit small plants in extreme alpine conditions because they will receive less reward in the process. Thus, differences in reproductive allocation with plant size may cause seed set and floral visitation in *T. caespitosum*.

Plant size is one of the major biotic factors that determined the amount of energy available for reproduction and seed development. Generally, large plants usually have large reproductive outputs (for example seeds or flowers) and have been confirmed in alpine plants (Rees & Venable, 2007; Sun et al., 2014; Venable & Rees, 2009). Large reproductive investment means more materials will be used to attract insects for pollination. Our results showed that the total number of flowers, the number of fruits, number of unseeded flowers, and reproductive allocation of *T. caespitosum* were clearly influenced by plant size (Figures 3 and 4; Table 1). He et al. (2008) found that the biomass of reproductive organs and allocation of resources to reproduction in three Oxytropis species from the Qinghai‐Tibet Plateau increased with increases in plant size. Similar positive relationships between reproductive allocation and plant size have also been found in *Polygonum macrophyllum* (Meng, Wang, Liu, & Zhu, 2011). Our results similarly showed that the total number of flowers, the number of fruits, number of unseeded flowers, and reproductive allocation were significantly increased (*p* < 0.05) with increases in plant size in two populations of *T. caespitosum* (Figures 4 and 5). This can be simply explained by the fact that larger *T. caespitosum* produced more reproductive biomass than smaller ones. This may be attributed to plant modular architecture, as relatively large individuals within a population have more vegetative and reproductive modules than smaller individuals (Weiner, 1988; Niklas, 1993). Underlying size‐dependent reproductive allocation is an adaptation of *T. caespitosum* to extreme environments. Reproductive allometry related to plant size is thought to be due to environmental variability and is interpreted as an adaptive strategy of plant growth and allocation (Bonser & Aarssen, 2009). Allocating more biomass to functions that maximize vegetative growth is more common under harsh environments (Shabir et al., 2017). For small individuals, large resource investment in reproduction may have a negative influence on future reproduction, growth, or survival (Obeso, 2002; Shibata & Kudo, 2016). Inversely, large resource investment in vegetative growth may lead to positive effects on future reproduction, especially in the harsh alpine environment where conditions (high altitude, low temperatures, overcast conditions, short growing season, unstable substrate, intense radiation, and relatively unpredictable weather and high winds) are not advantageous to plant survival. This type of allocation therefore implies trade‐offs for *T. caespitosum* in harsh alpine environments because resources allocated to one function or organ are not available to other functions or organs (Weiner, 2004).

In brief, flies are the main visitors of *T. caespitosum*, and floral visitors with the capacity to pollinate *T. caespitosum* over a broader temporal range (primarily nonbee taxa) have a stronger influence on *T. caespitosum* reproductive fitness than those with a more restricted temporal range. This is significant because it demonstrates how abiotic conditions select for particular pollinators, and how this may have a cascading effect on a long‐lived plant's fitness. Differences in reproductive allocation with plant size may cause seed set and floral visitation in *T. caespitosum*. Larger plants produce disproportionately more flower and fruit. This size‐dependent allocation of resources implies trade‐offs by *T. caespitosum* in harsh alpine environments and reflects cushions survival strategies.

## CONFLICT OF INTEREST

None declared.

## AUTHOR CONTRIBUTIONS

Ruiming Zhao designed and performed the study. Hua Zhang performed statistical analysis. Lizhe An conceived and financed this study. All the authors participated in writing the manuscript.

## Data Availability

We agree to make our data publicly available in a relevant repository.
